# Intracranial actinomycosis of odontogenic origin masquerading as auto-immune orbital myositis: a fatal case and review of the literature

**DOI:** 10.1186/s12879-019-4408-2

**Published:** 2019-09-02

**Authors:** G. J. Hötte, M. J. Koudstaal, R. M. Verdijk, M. J. Titulaer, J. F. H. M. Claes, E. M. Strabbing, A. van der Lugt, D. Paridaens

**Affiliations:** 1 Department of Ophthalmology, Erasmus Medical Center, Rotterdam, The Netherlands; 2 Department of Orbital Oculoplastic and Lacrimal Surgery, The Rotterdam Eye Hospital, PO box 70030, 3000 LM Rotterdam, The Netherlands; 3 Department of Oral and Maxillofacial Surgery, Erasmus Medical Center, Rotterdam, The Netherlands; 4 Department of Pathology, Erasmus Medical Center, Rotterdam, The Netherlands; 5 Department of Neurology, Erasmus Medical Center, Rotterdam, The Netherlands; 6 Department of Neurology, Franciscus Gasthuis and Vlietland, Rotterdam, The Netherlands; 7 Department of Radiology, Erasmus Medical Center, Rotterdam, The Netherlands

**Keywords:** Actinomycosis, Intracranial infection, Intraorbital infection, Odontogenic origin, Orbital myositis

## Abstract

**Background:**

*Actinomycetes* can rarely cause intracranial infection and may cause a variety of complications. We describe a fatal case of intracranial and intra-orbital actinomycosis of odontogenic origin with a unique presentation and route of dissemination. Also, we provide a review of the current literature.

**Case presentation:**

A 58-year-old man presented with diplopia and progressive pain behind his left eye. Six weeks earlier he had undergone a dental extraction, followed by clindamycin treatment for a presumed maxillary infection. The diplopia responded to steroids but recurred after cessation. The diplopia was thought to result from myositis of the left medial rectus muscle, possibly related to a defect in the lamina papyracea. During exploration there was no abnormal tissue for biopsy. The medial wall was reconstructed and the myositis responded again to steroids. Within weeks a myositis on the right side occurred, with CT evidence of muscle swelling. Several months later he presented with right hemiparesis and dysarthria. Despite treatment the patient deteriorated, developed extensive intracranial hemorrhage, and died. Autopsy showed bacterial aggregates suggestive of actinomycotic meningoencephalitis with septic thromboembolism. Retrospectively, imaging studies showed abnormalities in the left infratemporal fossa and skull base and bilateral cavernous sinus.

**Conclusions:**

In conclusion, intracranial actinomycosis is difficult to diagnose, with potentially fatal outcome. An accurate diagnosis can often only be established by means of histology and biopsy should be performed whenever feasible. This is the first report of actinomycotic orbital involvement of odontogenic origin, presenting initially as bilateral orbital myositis rather than as orbital abscess. Infection from the upper left jaw extended to the left infratemporal fossa, skull base and meninges and subsequently to the cavernous sinus and the orbits.

**Electronic supplementary material:**

The online version of this article (10.1186/s12879-019-4408-2) contains supplementary material, which is available to authorized users.

## Background

Most odontogenic infections are self-limiting and localized. In some cases however, they may cause a variety of complications [1, 2]. Infections can spread intracranially, leading to life-threatening complications such as brain abscess, meningitis or meningoencephalitis, orbital abscess and cavernous sinus thrombosis [1, 3].

In the order of *Actinomycetales*, *Actinomyces* is a genus of the *Actinomycetaceae* family, whereas *Nocardia* is a genus of the *Nocardiaceae* family. Both genera belong to the normal commensal flora of the oropharyngeal cavity and are known to rarely cause intracranial infection of odontogenic origin [4].

In this report we describe a fatal case of presumed intracranial and presumed intra-orbital actinomycosis of odontogenic origin. To our best knowledge, this specific case shows a presentation and clinical course not reported on before.

## Case presentation

A 58-year-old man first presented with pain in the left upper jaw. Medical history included polyarthrosis with secondary arthritis treated with hydroxychloroquine. After 2 weeks, the upper left second molar was extracted by his dentist. Three days later, routine blood examination by the rheumatologist showed a highly increased C-reactive protein (CRP) level, which was interpreted as a maxillary infection and treated with clindamycin for 5 days.

Six weeks later he experienced sudden diplopia and progressive pain in the left temporal/frontal region and behind the left eye. On Magnetic Resonance Imaging (MRI) of the brain and jaw region only a small uncomplicated lipoma near the parotid gland was found. He was admitted to the rheumatology department on suspicion of giant cell arthritis. Erythrocyte sedimentation rate (ESR) was normal, CRP was only mildly elevated and biopsy of the temporal artery was negative. Nonetheless, the pain and diplopia responded well to a three-day course of high dose intravenous steroids (1000 mg/day).

**Fig. 1:**
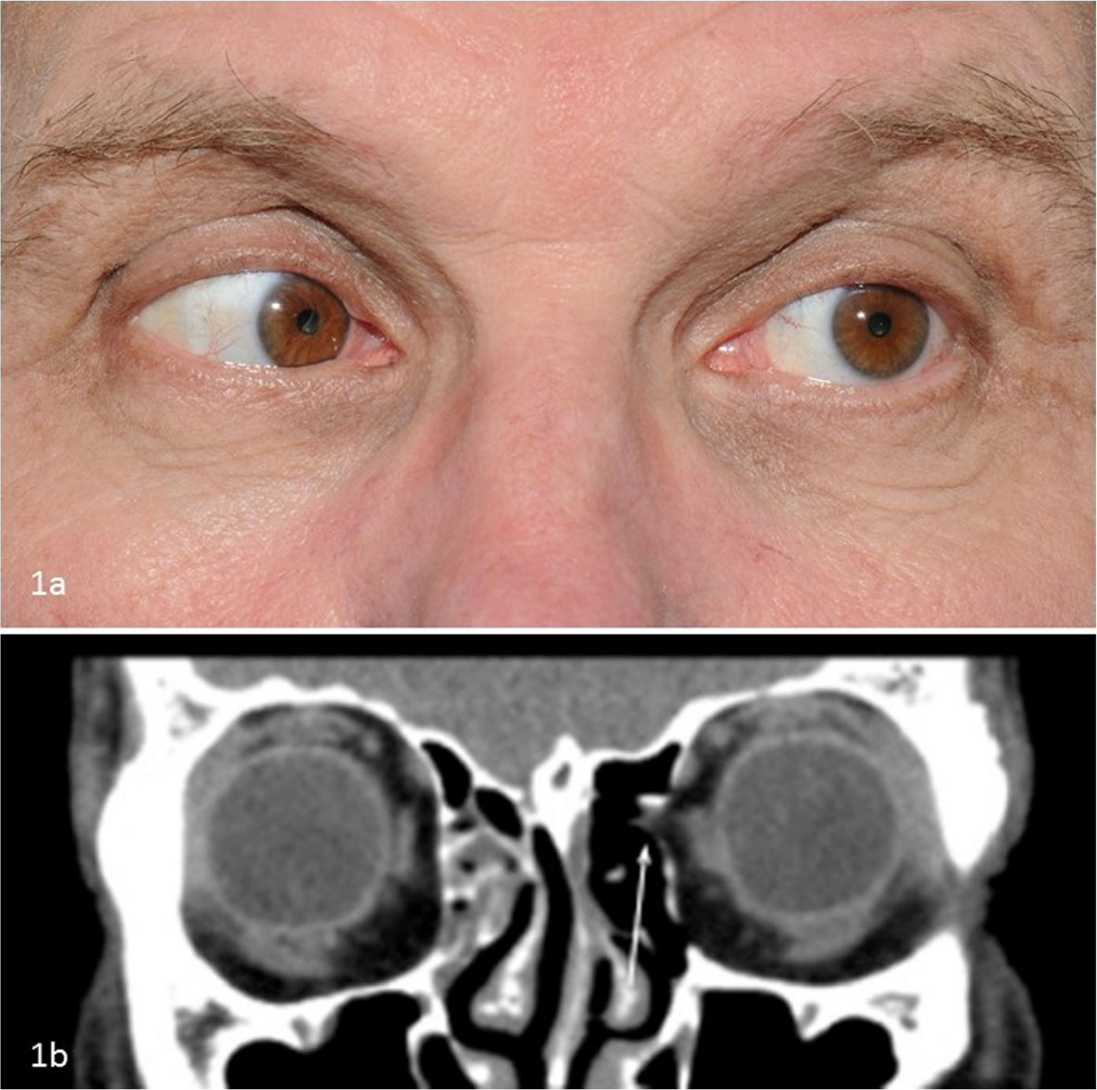
**a** Orthoptic evaluation shows an impaired abduction with over-elevation in adduction of the left eye, suggesting a mechanical component. **b** CT image (coronal reconstruction) which demonstrates a defect in the left lamina papyracea

Within a week after cessation of steroids he experienced an increase in pain and diplopia and was admitted to the neurology department for further evaluation. On neurologic examination, there was an abduction deficit but no signs of meningitis. Cerebral spinal fluid (CSF) was normal and MR Venography (MRV) showed no pathology of the dural venous sinuses. Serologic tests were negative for *Borrelia burgdorferi* and *Treponema pallidum* (Venereal Disease Research Laboratory test and Rapid Plasma Reagin test). Ophthalmic examination was unremarkable but orthoptic evaluation confirmed the abduction deficit with over-elevation in adduction of the left eye, suggestive of a mechanical component (Fig. 1a). Computed Tomography (CT) imaging of the orbit showed a defect in the left lamina papyracea, closely related to the left medial rectus muscle, with prolapse of orbital fat into the ethmoid sinus. Also, the medial rectus muscle was slightly enlarged (Fig. 1b). The findings were interpreted to be either an occult trauma to the medial orbital wall with reactive myositis, or an auto-immune orbital myositis. Oral steroids (60 mg initially) were prescribed and he was referred to the department of oral and maxillofacial surgery for evaluation. On examination, the extraction site of the upper left second molar was unremarkable and there were no complaints in that region. During surgical exploration of the left medial wall region there were no signs of infection or abnormal tissue for biopsy. The appearance of the bony defect corresponded well to the suspected traumatic cause and the medial wall was uneventfully reconstructed using a polydioxanone (PDS) sheet. Postoperatively the ocular motility improved and prisms were prescribed. The headaches, however, returned and he was referred back to the neurologist for further evaluation.

**Fig. 2:**
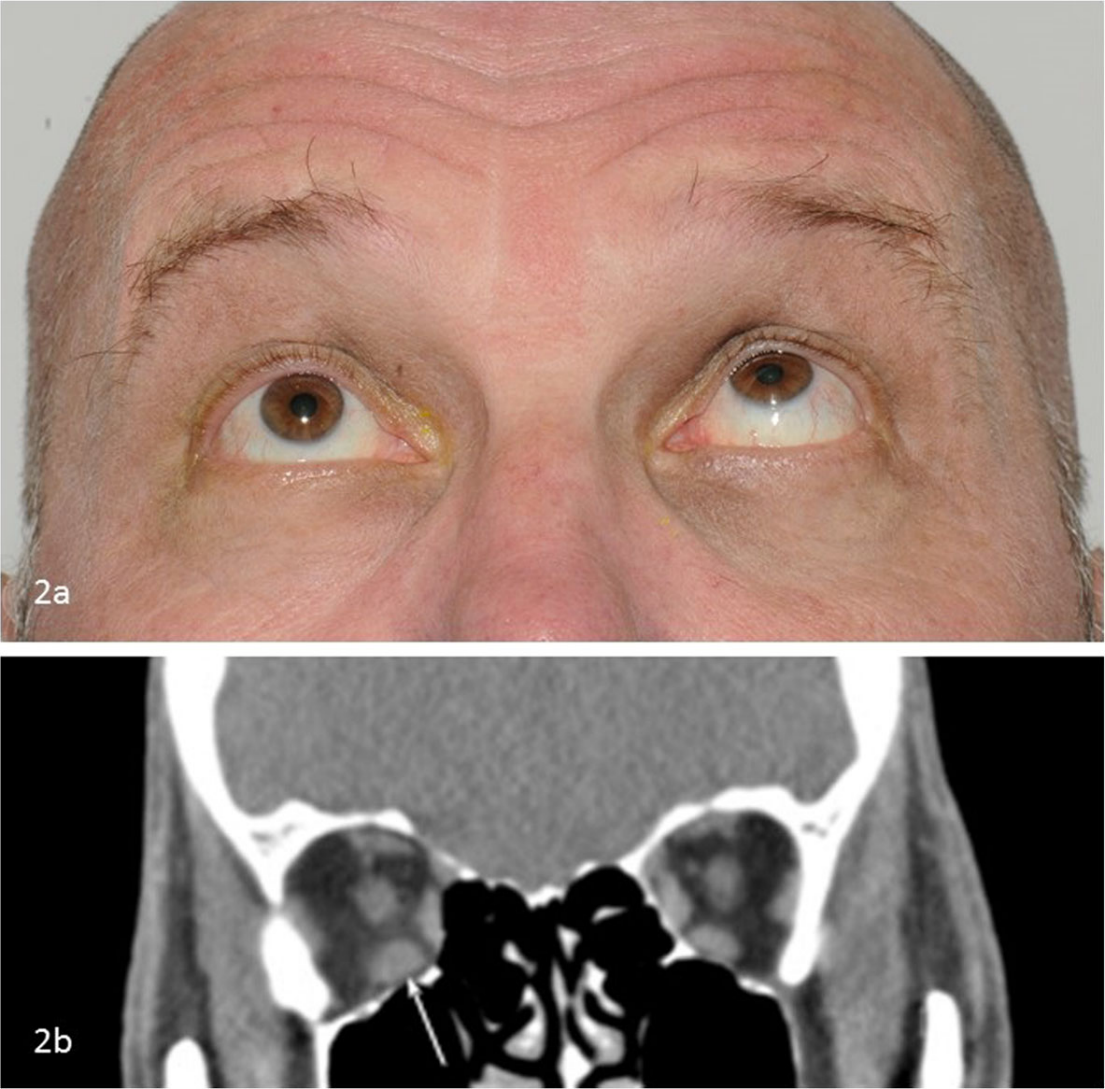
**a** Orthoptic evaluation now shows impaired elevation and abduction of the right eye. **b** CT image (coronal reconstruction) which shows enlargement of the right inferior rectus muscle with inflammatory fat infiltration surrounding the muscle

After 2 weeks the diplopia worsened as the steroids were tapered to 20 mg. In addition to the slight residual abduction deficit of the left eye, orthoptic evaluation now demonstrated impaired abduction and elevation of the right eye (Fig. 2a). Neurological examination was otherwise unremarkable. ESR and white blood cell (WBC) count were increased (58 mm/h and 23.7 × 10^9^/L respectively), while anti-nuclear antibodies (ANA) and anti-neutrophil cytoplasmic antibodies (ANCA) were negative. New MRI and CT scans were performed which showed enlargement of the inferior rectus muscle of the right eye, surrounded by an inflammatory fat infiltration (Fig. 2b). These findings fitted the previous suspected diagnosis of (auto-immune) orbital myositis. Steroid dosage was initially increased to 60 mg and then tapered.

Seven months after the initial presentation, he presented with a transient right hemiparesis and dysarthria. On a CT scan a slightly increased density in the suprasellar cistern was found but no signs of cerebral ischemia or hemorrhage. A Fludeoxyglucose Positron Emmision Tomography (FDG-PET) scan showed increased cerebral activity and at the skull base, but no evident vasculitis. One out of six blood cultures showed *Staphylococcus aureus* growth, which was considered to be a contamination.

**Fig. 3:**
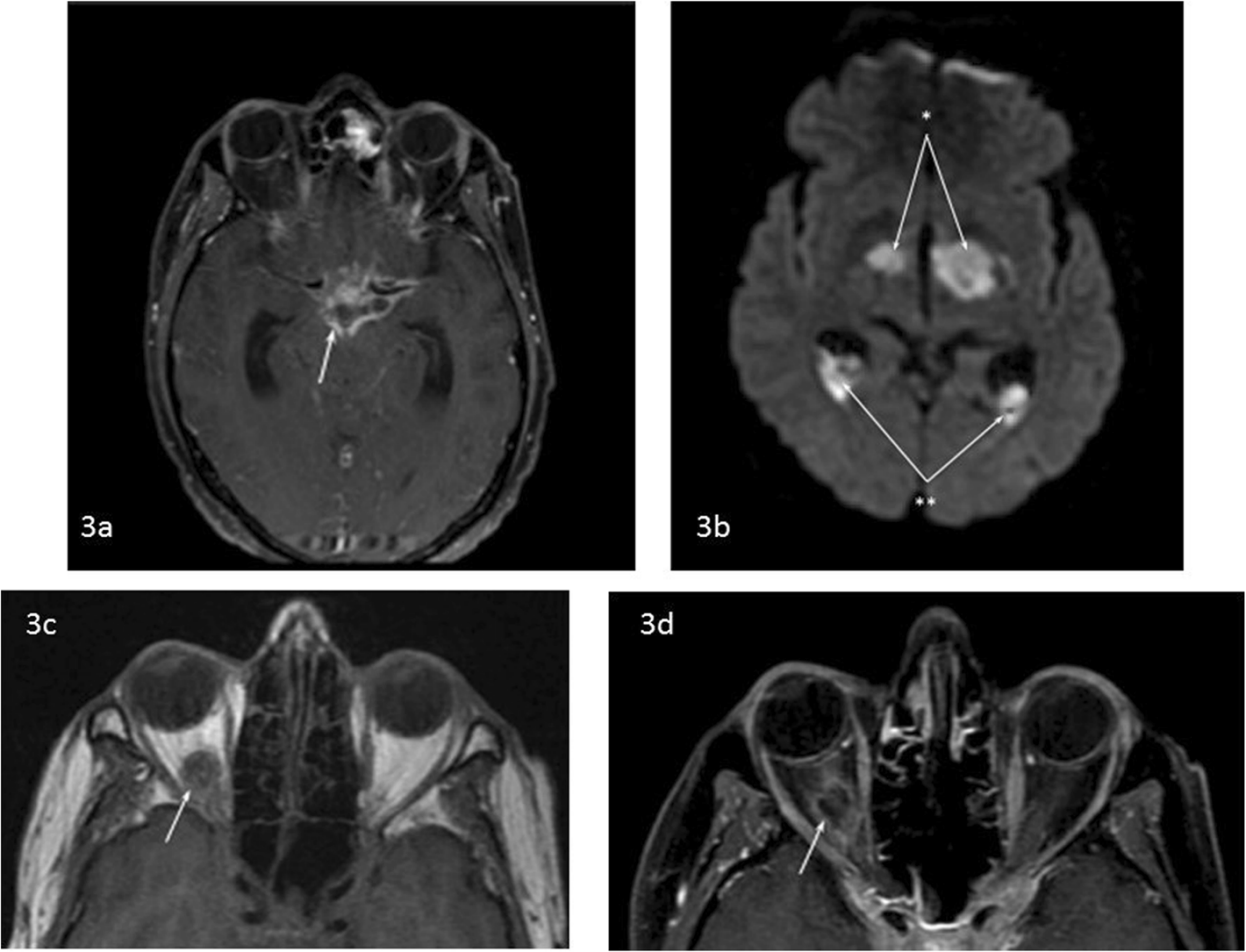
**a** T1w fat-suppressed post-gadolineum MRI scan which shows contrast enhancement at the surface of the basal brain structures compatible with basal meningitis (arrow). **b** Diffusion-weighted image shows high signal, which represents diffusion restriction caused by an acute brain infarct, bilaterally in the thalamus (arrow indicated by asterisk). In addition the high signal posterior in the ventricles is suggestive of ventricular empyema (arrow indicated by double asterisk). **c** and **d** Pre and post-gadolineum T1w MRI scan demonstrating a lesion in the right orbit with ring enhancement compatible with an abscess

After being transferred to the university hospital, further evaluation was initiated. Protein levels in the CSF were elevated 25 times the normal value and cytology showed signs of an acute inflammation with increased neutrophil levels, but no microorganisms could be identified through Gram stain and culture. On a new MRI scan, abnormalities at the skull base, cerebrum and brainstem were found, indicating basal meningitis, together with bilateral thalamic infarcts. Also it now showed a lesion in the right orbit, suggestive of an abscess (Fig. 3). The imaging results and the highly increased protein levels in the CSF suggested tuberculous meningitis. However, as he had recently travelled to Indonesia, other viral/bacterial aetiologies were considered and broad-spectrum antibiotics (amoxicillin and ceftriaxone) and aciclovir together with antimycobacterial therapy (isoniazid, rifampicin, pyrazinamide and ethambutol) and dexamethasone was commenced. The orbital abscess fitted the presumed diagnosis of tuberculosis but, more importantly, his poor clinical condition did not permit biopsy of the lesion for confirmation. Further evaluation for tuberculous meningitis was initiated. For both CSF and sputum, Auramine staining and polymerase chain reaction (PCR) for *Mycobacterium tuberculosis* were negative. Interferon-gamma release assay was uninterpretable. Bacterial, mycobacterial and fungal cultures were obtained from blood, CSF and sputum. Serologic testing for toxoplasmosis showed increased IgG-, but normal IgM-levels, together with negative PCR for *Toxoplasma gondii* in CSF, indicating an earlier exposure but no current infection. To rule out lymphoma, CSF samples showed no monoclonal B-cell or abnormal T-cell populations. Despite treatment the patient deteriorated. After 2 weeks there was an acute clinical deterioration and CT showed an extensive intracranial hemorrhage. All treatment was discontinued and he deceased shortly after (Additional file 1).

**Fig. 4:**
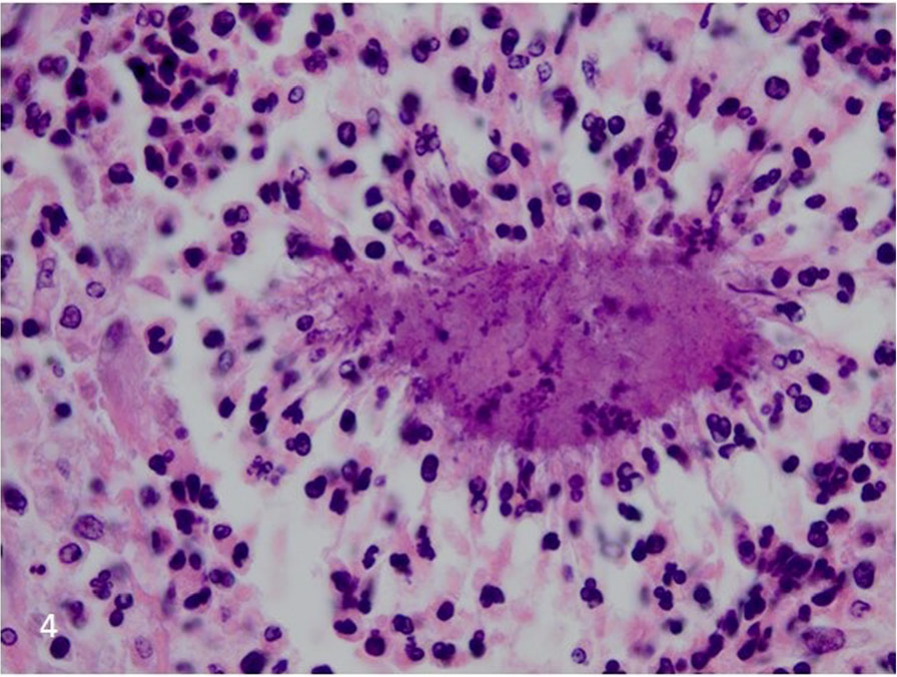
Autopsy samples from the brain showed gram-positive filamentous microbes

Post-mortem the mycobacterial cultures of CSF, sputum and blood were negative.16S rRNA PCR returned positive for the CSF samples, but further sequencing was uninterpretable. During autopsy there were no signs of mycobacterial infection. There were, however, signs of meningoencephalitis with vasculitis and septic thromboembolism based on gram-positive filamentous microbes such as *Actinomyces* or *Nocardia* (Fig. 4). Further distinction between the two could not be made histologically and the paraffin embedded specimens allowed no further genetic determination by sequencing. Unfortunately, the autopsy application lacked information on the orbital lesions and as such this region was not explored during the autopsy.

**Fig. 5:**
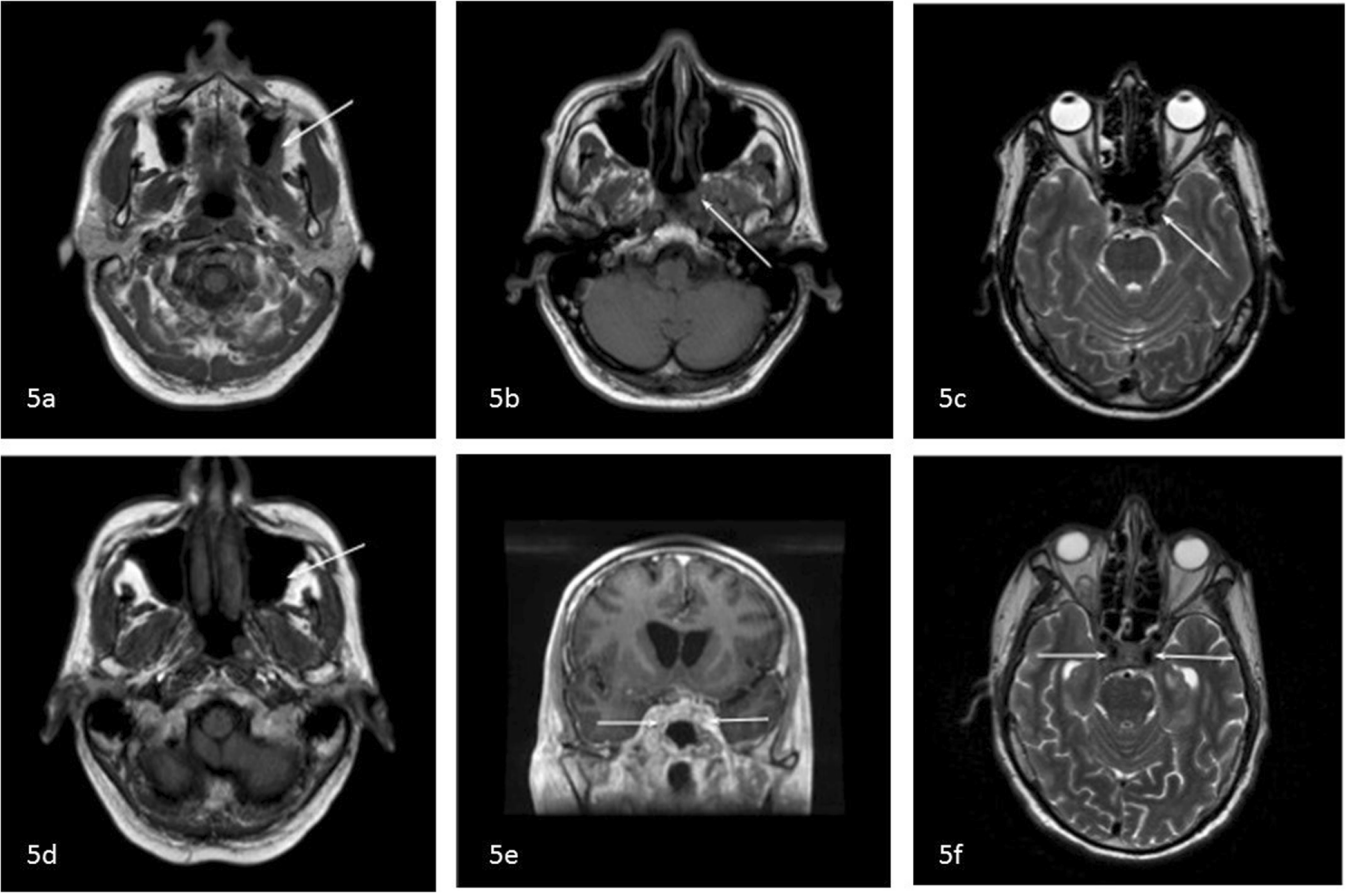
Reexamination of imaging studies. **a**–**c** *T1w* MRI scans from 2 months after the initial presentation show (**a**) a soft tissue mass in the left infratemporal fossa (arrow), with (**b**) decreased signal intensity in the left central skull base (arrow) and (**c**) a low signal intensity in the left cavernous sinus (arrow). **d**–**f** T1w MRI scan performed at a later stage revealed (**d**) resolution of the lesion in the infratemporal fossa (arrow), but there is bilateral enlargement of the cavernous sinus (arrows) and involvement of the pituitary gland on the coronal post-gadolinium T1w scan (**e**) and the T2w Scan (**f**)

All imaging studies were reexamined. In two of the MRI scans (performed 2 months after the initial presentation) a soft tissue mass was now identified in the left infratemporal fossa, together with subtle abnormal signal intensities at the left skull base and the left cavernous sinus (Fig. 5a–c). In an MRI scan performed at a later stage, the abnormal intensity at the infratemporal fossa and skull base was less evident, but there was bilateral enlargement of the cavernous sinus and involvement of the pituitary gland (Fig. 5d–f). We concluded that the intracranial and intraorbital actinomycotic infection appeared to have originated from an odontogenic maxillary infection.

## Discussion and conclusions

*Actinomyces* and *Nocardia*, belong to the normal commensal flora of the oropharyngeal cavity [4–6]. Although the name *Actinomyces* translates to “ray fungus”, these pathogens are in fact branching filamentous prokaryotic bacteria related to mycobacteria [4, 5, 7, 8]. Although some are microaerophilic, most *Actinomyces* species require strict anaerobic conditions to grow, whereas *Nocardia* grows best in aerobic conditions [5, 8]. Although this patient used hydroxychloroquine, *Actinomycetes* do not appear to have a predilection toward immunocompromised patients. They have low virulence but can lead to actinomycosis if there is disruption of the mucosal barrier [4, 5].

As outlined by Van Dellen, actinomycosis can disseminate intracranially through direct invasion, along fascial planes and by extension through the base of the skull or meninges. Also, it can spread through perineural extension or by hematogenous route [4]. Although the first report of intracranial actinomycotic infection dates back to as early as 1882 by Ponfick, it is in fact a very rare finding (3% of actinomycotic cases) [4, 5].

Five different orbital complications of odontogenic infection have been outlined by Allan et al.: preseptal cellulitis, orbital cellulitis, orbital subperiosteal abscess, orbital abscess and cavernous sinus thrombosis [9, 10]. Although an orbital abscess was present at a later stage, this is the first report of actinomycotic orbital involvement presenting initially as an orbital myositis.

Moreover, while bilateral cavernous sinus involvement has been reported, the route of dissemination in this particular case is of interest. The infection presumably started near the upper left second molar and extended to the left infratemporal fossa, skull base and meninges. Next, infection must have spread to the left cavernous sinus, the sellar region and subsequently to the right cavernous sinus. The cavernous sinus is connected with nearby regions (such as the maxillary dentition) by valveless veins, allowing infections to spread bidirectionally [1, 11]. As such, the infection extended from the cavernous sinuses to the left and subsequently right orbit, evidenced by the muscle enlargement, fat infiltration and, later, orbital abscess. Involvement of the cavernous sinus first, with subsequent retrograde bilateral spread to the orbits has not yet been described in actinomycosis. An uncertainty in our report is the fact that, unfortunately, the orbits were not explored during autopsy. As such, actinomycotic involvement of this region could not be confirmed. The relationship between the defect in the lamina papyracea and the orbital myositis is unclear. The medial rectus inflammation might have weakened the thin medial wall, making it more fragile, causing fracture at rubbing, but this is speculative. It could well have been a chance finding, which clouded the initial diagnostic process.

Few case reports describe painful ophthalmoplegia, characterized by periorbital pain and paralysis of the oculomotor nerves, as the presenting sign of actinomycosis with cavernous sinus involvement [12–14]. In our case however, despite cavernous sinus involvement, the restriction of ocular motility seemed not paralytic but rather mechanical or restrictive in nature. This raises the question as to why bilateral involvement of the cavernous sinus did not lead to apparent clinical or radiological signs of orbital congestion or evident paralysis of the oculomotor nerves. Possibly, the cavernous sinus infection resulted from direct extension from the infratemporal regions, rather than being caused by infected thrombi [11]. The eventual cause of death was shown to have been the intracranial hemorrhage, which resulted from vasculitis and thromboembolism, together with meningoencephalitis. Koda et al. describe a case of actinomycotic meningitis in which secondary necrotizing arteritis lead to the perforation of a pseudoaneurysm and subsequent subarachnoid hemorrhage [15]. In fact, odontogenic infections have been shown to be involved in the pathogenesis of ruptured cerebral aneurysms [16, 17].

Retrospectively, the history of maxillary pain and possible molar infection should have been emphasized more. Possibly, the recognition of the infection was hampered by several obscuring clinical findings. Furthermore, unlike in the patient reported by Sullivan there were no apparent infectious foci for biopsy until late stages of the disease [18]. In addition, subtle radiological features that pointed at disease were overlooked and only diagnosed at a late stage.

From an ophthalmological point of view, at first it appeared to be a clinically apparent case of idiopathic or reactive orbital myositis; i.e. enlargement of the extraocular muscles and tendons with surrounding infiltration of the orbital fat and corresponding painful impairment of ocular motility. As idiopathic orbital myositis is a *diagnosis per exclusionem*, the question is raised as to when one can assume an infection is sufficiently excluded. Clinical and radiological findings in infectious myositis are similar to those of auto-immune myositis [19] and initially CRP, ESR, WBC and CSF were not suggestive of infectious disease. Although there is no consensus on diagnostic criteria for idiopathic orbital myositis, the above-mentioned clinical features are in line with what can be expected in this disease entity. The fact that it responded well to steroid therapy strengthened this assumption [20]. Mombaerts et al. state that many orbital disorders respond well to steroids initially and thus is a weak tool in identifying the cause of orbital inflammation. Therefore they advocate early tissue biopsy to exclude other causes, with the exception of lesions located in the orbital apex or extraocular muscles [21]. .For orbital myositis, the initial treatment of choice mostly is a trial of steroids, while biopsies are done in non-responsive patients or those presenting with clinical recurrence. In line with these guidelines, we performed surgical exploration of the affected area in the left orbit as the diplopia recurred after cessation of steroids. However, no abnormal tissue was found for biopsy. It is likely that the courses of steroids have supported the dissemination of the infection [12]. .Also, it is questionable if the PDS sheet that was used could have served as a foreign body that has aggravated the infection.

At a later stage, the findings of the CSF and imaging led us to believe it could be tuberculous meningitis and, although broad-spectrum antibiotics and aciclovir were initiated also because of a recent travel history, antimycobacterial treatment was emphasized alike. At this stage imaging also showed an abscess in the right orbit, which fitted the presumed diagnosis of tuberculosis. More importantly, however, his clinical condition did not permit biopsy of the lesion for confirmation. It is likely that this orbital abscess would have evolved in an earlier stage if steroids had not been prescribed for a prolonged period of time. If so, his condition at that time probably would have permitted biopsy of the lesion, allowing for adequate treatment early into the clinical course. Unfortunately also, the broad range 16S rRNA PCR in the CSF did not return positive until after death, confirming the presence of bacteria.16S rRNA PCR is rarely positive in tuberculous meningitis and, in fact, *Mycobacterium tuberculosis* has been used as a negative control to measure specificity of the assay [22]. As such, earlier availability of this result would have questioned the diagnosis. An uncertainty in our report is the fact that, due to poor quality DNA derived from the formalin fixated paraffin embedded obduction sample, sequencing for the genetic subclassification of the gram-positive filamentous microbes was not possible. Additional targeted 16S rRNA assays, in order to distinguish *Actinomyces* from *Nocardia,* turned out negative due to poor quality of the RNA.

Actinomycosis is characterized by a long duration of mild, nonspecific, constitutional symptoms with or without fever; a true silent assassin [4, 23]. Van Dellen states that actinomycosis should be considered based on several features: (1) chronicity, (2) progression across tissue boundaries, (3) masslike features, (4) disease features that resolve and recur, and (5) refractory or relapsing infection after a short course of therapy [4].

Even when suspected based on the above mentioned features, actinomycosis is notably difficult to diagnose [4]. Although not specific to actinomycosis, imaging can show abnormalities such as cerebral and/or orbital abscess, meningitis and subdural/epidural empyema [4]. Interestingly, Sato et al. recently described FDG-PET scan findings identifying an odontogenic infection as the cause of a brain abscess [24] Unfortunately, in our case no such findings were seen on the FDG-PET scan. *Actinomycetes* are extremely difficult to culture [5, 25]. It is therefore best to use PCR techniques in addition to cultures. Although this does not test for antibiotic susceptibility, it could aid to alter empiric antibiotic coverage [25]. In our case, the results were indeed positive but unfortunately only after death. Besides 16S rRNA PCR, Wilson et al. recently suggested metagenomics to identify the right pathogen [26]. Eventually, diagnosis is often made histologically, showing sulfur granules, which are composed of aggregates of microbes, often featuring the Splendore-Hoeppli Phenomenon, and contain calcium phosphate [23].

After establishing the diagnosis, the treatment of actinomycosis poses an even bigger challenge. High dose intravenous penicillin or amoxicillin for a prolonged period of time is the treatment of choice, although tetracyclines and clindamycin can be used as alternatives [4, 27]. At initial presentation, clindamycin was prescribed but only shortly and the specific species could have been resistant to clindamycin [27]. Moreover, clindamycin does not cross the blood-brain barrier making it impractical for the treatment of intracranial actinomycosis. At a later stage we commenced amoxicillin as part of broad-spectrum coverage. Although penicillin resistance is almost nonexistent, the vasculitis leading to the eventual fatal hemorrhage probably was too well advanced at this point [8, 27]. Also, *Actinomyces* aggregates into tight polymicrobial rosettes thereby preventing antibiotics from reaching the microbes within the aggregate. This further provides resistance against phagocytosis and accommodates ideal anaerobic conditions for growth [5, 25]. As such successful antibiotic treatment is difficult and surgical drainage is often required, if feasible [4, 23].

In conclusion, this is the first report of probable actinomycotic orbital involvement of odontogenic origin, presenting initially as an orbital myositis. Intracranial actinomycosis is a rare complication, notoriously difficult to diagnose and treat, with potentially fatal outcome. Imaging is aspecific and cultures are clouded by high false-negative results. Therefore, it is imperative to vigorously explore all recent medical history as this may be the only clue leading to the right diagnosis. When such rare infection is included in the differential diagnosis and the clinician is aware of the potential mode of spread, the cavernous sinus location of infectious masses would be more readily identified, thus allowing for earlier adequate treatment. Moreover, one should remain aware of the fact that idiopathic orbital myositis is a *diagnosis per exclusionem*. Therefore, an accurate diagnosis can only be established by means of histology and biopsy should be performed whenever feasible.

## Additional file

Additional file 1: Timeline of the relevant data. (PNG 132 kb)

## Data Availability

N/A

## Abbreviations

ANA: Anti Nuclear Antibodies
ANCA: Anti Neutrophil Cytoplasmic Antibodies
CRP: C-reactive Protein
CSF: Cerebrospinal Fluid
CT: Computed Tomography
ESR: Erythrocyte Sedimentation Rate
FDG-PET: Fludeoxyglucose Positron Emission Tomography
MRI: Magnetic Resonance Imaging
MRV: Magnetic Resonance Venography
PCR: Polymerase Chain Reaction
PDS: Polydioxanone
rRNA: Ribosomal Ribonucleic Acid
WBC: White Blood Cell
