# Supplementary figures and images for: Intracranial actinomycosis of odontogenic origin masquerading as auto-immune orbital myositis: a fatal case and review of the literature
Source: BMC Infect Dis. 2019 Sep 2;19:763. doi: 10.1186/s12879-019-4408-2 (PMC6720412; doi:10.1186/s12879-019-4408-2)

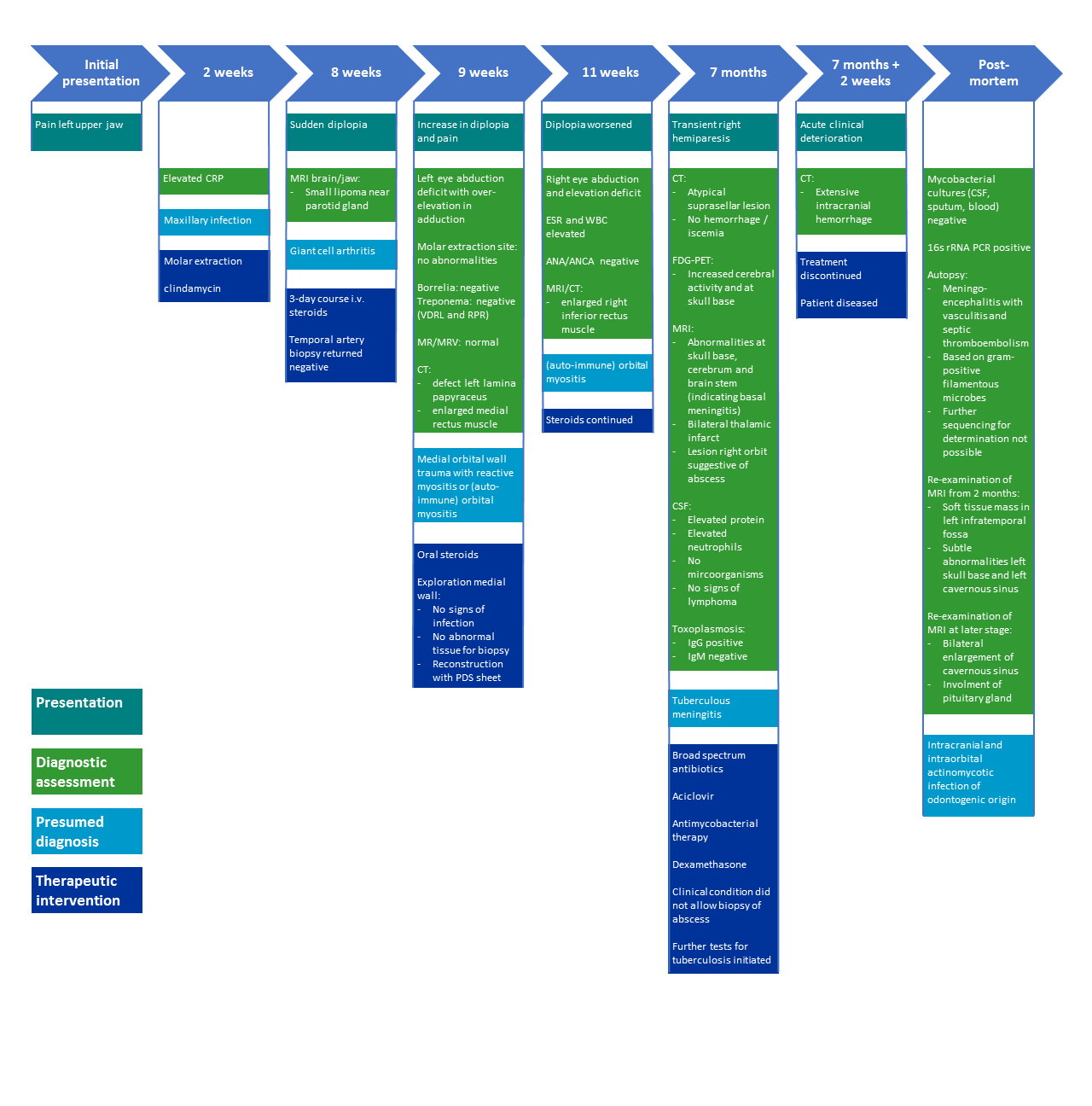

Supplement: Supplementary file 1 — Timeline of the relevant data. (PNG 132 kb) [file 12879_2019_4408_MOESM1_ESM.png]
